# Analysis of exosomal competing endogenous RNA network response to paclitaxel treatment reveals key genes in advanced gastric cancer

**DOI:** 10.3389/fonc.2022.1027748

**Published:** 2022-10-25

**Authors:** Jun Lei, Guifeng Zhang, Deyu Li, Jiangming Zhong, Qiao Chen, Li Lin, Zhenhua Liu

**Affiliations:** ^1^ Department of Pathology, Cangshan Hospital of the 900 Hospital of Joint Logistics Support Force, Fuzhou, Fujian, China; ^2^ Department of Oncology, Shengli Clinical Medical College of Fujian Medical University, Fujian Provincial Hospital, Fuzhou, Fujian, China

**Keywords:** biomarker, advanced gastric cancer, exosome, lncRNA, ceRNA network

## Abstract

**Background:**

Exosome is an important component of the tumor immune microenvironment and plays critical role in cancer pathogenesis. The exosome transcriptome of gastric cancer (GC) response to paclitaxel chemotherapy has not been investigated in the past.

**Methods:**

ceRNA microarrays were performed in exosomes from six advanced GC patients before and after paclitaxel treatment. Bioinformatics tools were used to identify differential expressing genes and construct competing endogenous RNA (ceRNA) networks. The importance of hub genes in the ceRNA network was confirmed by survival analysis and functional analysis.

**Results:**

A total of 213 differential mRNAs, 370 lncRNAs, and 376 circRNAs were identified, and hub genes in ceRNA networks were screened. The differential genes were associated with GO terms SNAP complex, gap junction, protein transporter activity, cytokine receptor, and KEGG pathways synaptic vesicle cycle, propanoate metabolism, Epstein–Barr virus infection, heparin, and steroid biosynthesis, and beta-alanine metabolism. ULK2, CYP2R1, BTLA, and miR-105-5p are prognostic genes for overall survival. Paclitaxel may target ULK2 which is involved in mitosis and cell cycle. miR-105-5p may target ULK2 3’UTR.

**Conclusion:**

The work for the first time identified exosomal RNA biomarkers and constructed a ceRNA network in GC response to paclitaxel, revealed novel molecular mechanisms of GC, and provided new candidates for GC diagnosis and treatment.

## Introduction

Gastric cancer (GC) is the fifth most common cancer and ranks as the third leading cause of cancer−associated death worldwide (1). Due to its frequently advanced stage at diagnosis, the majority of patients lose the opportunity of radical surgery, and chemotherapy remains the main strategy (1). With the development of modern biology techniques, molecular targets were discovered and applied in the diagnosis, treatment, and prognosis of tumors (2). As yet, it is difficult to predict whether targeted therapy will be effective for GC, and the underlying mechanisms of the diseases and drugs remain a mystery. To better understand the pathogenesis of GC and drug mechanisms of action (MoA), it is crucial to explore novel mechanisms of anti-GC drugs and provide new biomarkers.

Most eukaryotic cells secrete exosomes as extracellular vesicles that facilitate communication between them (3). Exosomes contain proteins, DNA, microRNAs, long noncoding RNAs, circular RNAs, etc., which are crucial for regulating tumor growth, metastasis, and angiogenesis during the cancer development process, and can serve as prognostic markers and/or grading bases for patients with tumors (3). RNAs longer than 200 nucleotides, referred to as long non-coding RNA (lncRNAs) are extensively transcribed from the genome but do not function as functional proteins. The lncRNAs are involved in multiple processes, including chromatin remodeling, genome integrity, RNA stabilization, and transcriptional regulation (4). The circular RNA family (circRNAs) are a type of noncoding RNA with a circular structure that regulates RNA transcription, interacts with proteins, acts as microRNA sponges, and sometimes can be translated into proteins (5). Small RNAs (miRNAs) bind to complementary sequences in the target mRNA and either inhibit translation or trigger degradation (6). mRNA translation may be controlled by competing endogenous RNAs (ceRNAs) such as lncRNAs and circRNAs. ceRNAs network contains interaction between lncRNAs, circRNAs, miRNAs, and mRNAs, and provides insight into the molecular pathogenesis of GC as well as a new direction for therapeutic approaches (7). This makes exosomes an ideal model for studying ceRNA networks and crosstalk among RNAs (8). However, the expression profiles of GC-derived exosomal ceRNAs and their potential functions have been largely unexplored. Hence, it is worth exploring the underlying molecular mechanisms and ceRNA targets for GC diagnosis and treatment.

Paclitaxel (Taxol) is a chemotherapy drug that has been used for the treatment of many different types of cancer (9). Microtubule-stabilizing drugs like paclitaxel have been approved by the Food and Drug Administration to treat ovarian, breast, and lung cancers, as well as Kaposi’s sarcoma (9). Paclitaxel monotherapy produced considerable improvement in tumor response and prognosis in GC (10). Depending on its structure and chemical properties, paclitaxel may have different mechanisms of action. Recently, paclitaxel has been repurposed for the treatment of renal interstitial fibrosis (11). Inhibition of STAT3 nucleus translocation by paclitaxel was demonstrated by disrupting STAT3’s interaction with tubulin (11). However, the transcriptome alteration and ceRNA network are not investigated in the paclitaxel-treated GC exosomes. Due to limited research, the anti−tumor effect and mechanisms of action (MoA) of paclitaxel in GC exosomes have remained elusive.

We also used bioinformatics methods to characterize the relationships among lncRNAs, circRNAs, miRNAs, and mRNAs. The differential expression profiles were detected by using microarray analysis. A ceRNA network was constructed. We identified hub genes, some of which were immune and cell cycle-related genes that are associated with patient survival. Furthermore, we infer the potential disease mechanism and paclitaxel MoA. Our research suggests that lncRNA/circRNA plays a key role in paclitaxel-induced exosome transcriptome alteration, and may provide a panel of key genes as targets for developing related therapeutic drugs in the future.

## Materials and methods

### Clinical specimens

A total of 12 plasma (2 mL) specimens were collected in this research. Overall, 6 plasma samples were derived from advanced GC patients (4 female and 2 male) with metastasis admitted to the Shengli Clinical Medical College of Fujian Medical University, Fujian Provincial Hospital, China from June to December in 2019. The average age of the patient was 61.5 ± 11.5. None of the patients had undergone radiotherapy, endocrine therapy, chemotherapy, or surgery before blood sample collection. Tissue biopsies were performed for histological confirmation. Additional 6 human plasma samples were derived from the same patients after two weeks of paclitaxel treatment. Written consent was provided by all the patients, and the Ethics Committee of Fujian Provincial Hospital approved all aspects of this study (ethics review number: K2022_03_101).

### Exosome isolation, RNA isolation, and transcriptome profiling

Plasma exosomes were collected using ExoQuick kit (System Biosciences Inc., CA, USA) following the manufacturer’s protocol. Briefly, ExoQuick Solution was added to plasma, and samples were incubated at 4°C overnight. The mixture was then centrifuged at 1,500 *g* for 30 min and the supernatant was removed by aspiration. Exosome pellets were collected for downstream analysis.

Total RNA was extracted with Trizol (Invitrogen) reagent and was quantified using a NanoDrop ND-2000 (Thermo Scientific), and was checked for RNA integrity by an Agilent Bioanalyzer 2100 (Agilent Technologies). The purity of total RNA will affect the labeling efficiency of probes and the results of chip hybridization, so total RNA was purified using QIAGEN RNeasy Kit. Take 250 ng of purified Total RNA for labeling and amplification. First, the AffinityScript-RT kit and Promoter Primer were used to reverse-transcribe RNA into the first strand of cDNA, then Anti-sense Promoter was used to generate the second strand of cDNA, T7 RNA polymerase was added, and the second strand of cDNA was amplified to generate cRNA. It was then labeled with the fluorescent dye Cyanine-3-CTP (Cy3) and purified using the QIAGEN RNeasy Kit. Finally, rolling hybridization was performed on SBC human ceRNA array V1.0 (Agilent Technologies) at 65° C for 17 h, and the original image was obtained by scanning with an Agilent Scanner G5761A (Agilent Technologies) after elution. Therefore, a total of 12 individual exosome samples were used for ceRNA array analysis.

### Differential RNAs screening and functional enrichment analysis

Feature Extraction (v10.7, Agilent Technologies) was used to extract expression data from raw chip images, followed by quantile normalization and differential analysis in the Genespring (v4.8, Agilent Technologies). Data was filtered after normalization, and only genes with at least 50% detection in all samples were kept for downstream analysis. The clusterProfiler package was used for enrichment analysis of the gene list (12). Gene Ontology (GO), Kyoto Encyclopedia of Genes and Genomes (KEGG), and Medical Subject Headings (MeSH) are used to map genes to known functional information sources. The overrepresentation of a term is defined as a P value with an adjustment for multiple testing.

### ceRNA network construction and hub genes identification

According to the hypothesis of the ceRNA mechanism, the over-lapped regions of the miRNA seed sequence binding sites both on differential lncRNAs/differential circRNAs (DEL/DEC) and differential mRNAs (DEM) were searched to predict DEL/DEC-miRNAs-DEM interactions with the software miRanda (version 3.3a, http://www.microrna.org) and miRDB (http://mirdb.org/) (13, 14). The regulatory network was visualized by Cytoscape (15). Hub genes of the ceRNA network were identified by the plugin NetworkAnalyzer.

### Survival analysis

Survival analysis was conducted in R 4.0.3 with survival and survminer packages (16). For NCBI GEO and TCGA datasets (17, 18), log-rank tests were used to analyze survival differences between patient groups with high/low hub gene expression or immune cell proportion. A significant difference in survival time was set at P <0.05.

### Protein-ligand docking analysis

The molecule structure for ULK2 was downloaded from the protein data bank (PDB) under the accession 6QAU. Paclitaxel structure was downloaded from the PDB Ligands database under the accession TA1. Protein-ligand docking was performed by SwissDock (19, 20). Protein-ligand complex hydrogen was predicted by Protein Plus (21). PoseView was used to generate two-dimensional diagrams of complexes to visualize hydrogen bonds (22).

### Statistical analysis

One-way ANOVA was used to detect the difference in means among groups. Paired Student’s t-test was used to compare the means of gene expression before and after drug treatment. Statistical significance was set at 0.05 unless otherwise specified. The correlation analysis for two genes was performed by calculating the Pearson correlation coefficient.

## Results

### Differentially expressed exosomal lncRNAs, circRNAs, and mRNAs in advanced gastric cancer patients treated with paclitaxel

**Figure 1:**
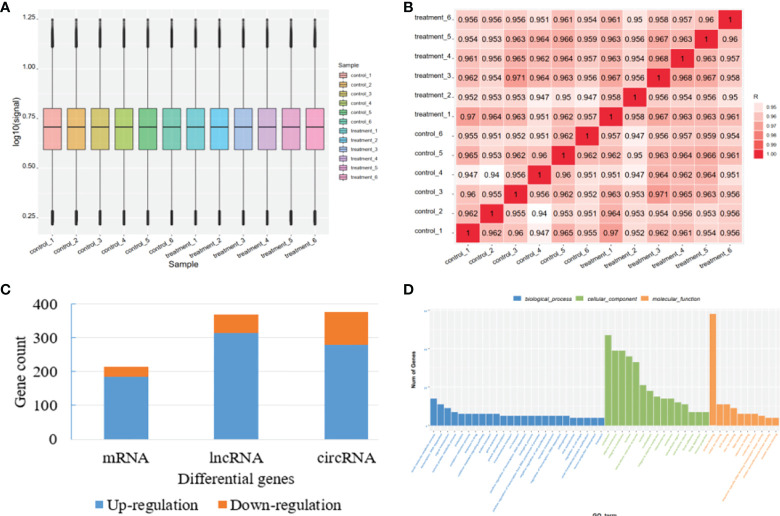
Quality control for microarray data and overview of the differential genes. **(A)** RNA expression boxplot. **(B)** Global gene expression correlation analysis across samples. **(C)** The number of differential genes in mRNA, lncRNA, and circRNA. **(D)** Differential gene count in different GO categories.

**Figure 2:**
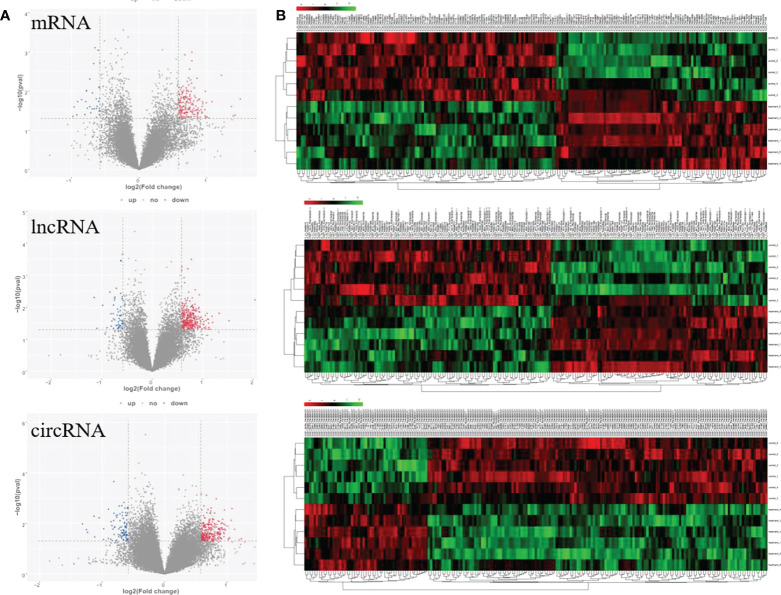
Volcano plots and clustering heat map for significantly differential genes response to paclitaxel treatment. **(A)** Volcano maps for significant mRNA, lncRNA, and circRNA. **(B)** Heat map for significant mRNA, lncRNA, and circRNA.

To fully understand the mechanism of paclitaxel, we simultaneously analyzed the profiles of DEL, DEC, and DEM through microarray. RNA expression boxplot and global gene expression correlation analysis across samples indicated the quality of our data is good (**Figures 1A, B**). Genes with |fold change|≥ 1.5 and P < 0.05 are defined as differentially expressed genes. A total of 961 differential genes were identified with different GO terms (**Figures 1C, D**). In the paclitaxel treatment group, 215 mRNAs (184 up-regulated and 31 down-regulated), 370 lncRNAs (315 up-regulated and 55 down-regulated), and 376 circRNAs (280 up-regulated and 96 down-regulated) were identified with significantly differential expression (**Figure 2A**). The volcano map and hierarchical cluster analysis of lncRNA, circRNA, and mRNA showed significant differences of exosomal genes between the control group and paclitaxel treatment group (**Figure 2B**).

### Functional enrichment analysis of differentially expressed RNAs

**Figure 3:**
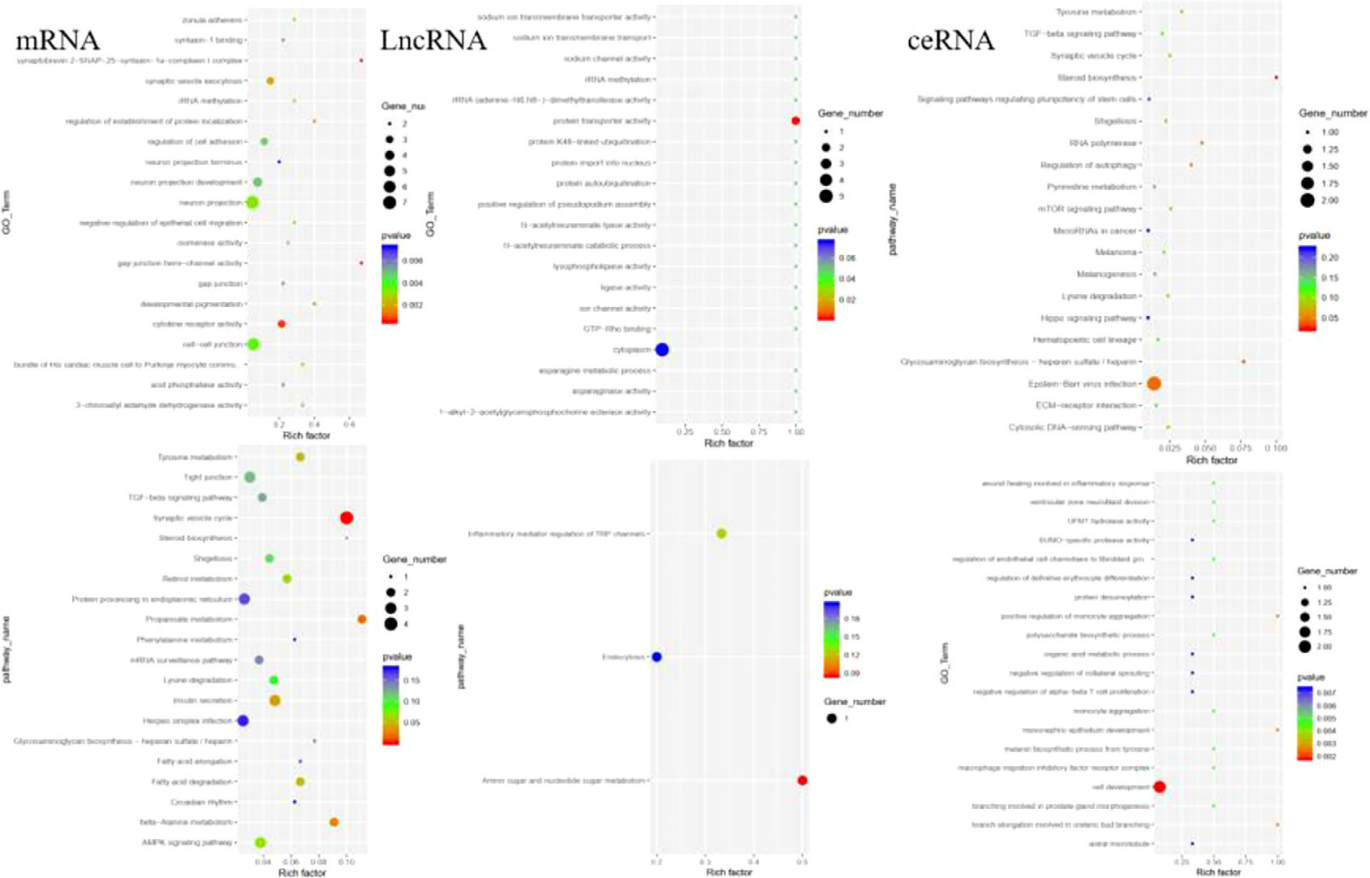
GO and KEGG analysis revealed the potential mechanism of DEMs, DELs, and DECs in paclitaxel treatment.

The functions of DELs, DEMs, and DECs were analyzed by GO and KEGG. The GO terms in DEMs are related to SNAP complex, gap junction, and cytokine receptor. KEGG analysis revealed the potential mechanism of DEMs in paclitaxel treatment, including synaptic vesicle cycle, propanoate metabolism, and beta-Alanine metabolism. The GO terms in DELs target genes are mainly related to protein transporter activity. The GO analysis of DECs showed that the functional predictions of target genes were mainly enriched in Epstein–Barr virus infection, heparin, and steroid biosynthesis (**Figure 3**). KEGG analysis suggested the potential mechanism of DECs in paclitaxel treatment (**Figure 3**). DECs participates in the inflammatory response, monocyte aggregation, and mesonephric epithelium development.

### Correlation analysis of lncRNA–mRNA pairs

**Figure 4:**
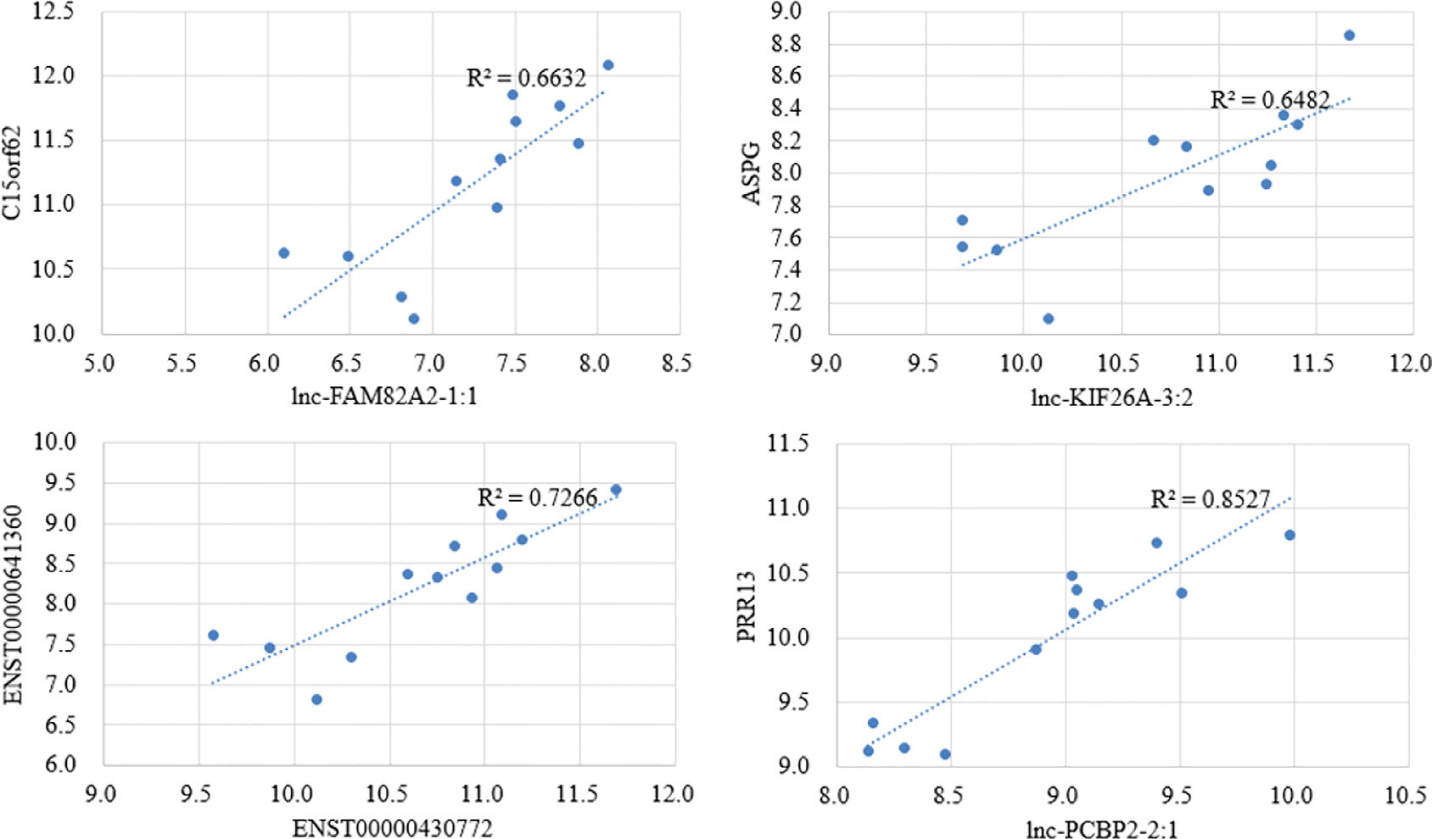
Scatter plots for the top 4 highly correlated lncRNA–mRNA pairs by correlation analysis.

There are a variety of ways in which lncRNA regulates biological processes. Direct interaction with mRNA can change chromatin’s spatial conformation, which, in turn, influences the expression level of mRNA. Thus, we performed expression correlation analysis to identify potential regulatory relationships. We analyzed those lncRNA-mRNA pairs with high correlation values (|R|>0.8) and found that the lncRNA target genes were enriched with nuclear replication fork (P =0.04), suggesting the potential mechanism of lncRNA in regulating mRNA expression. Some of the differential pairs are shown in **Figure 4**. Interestingly, the association of lnc-KIF26A and ASPG phenotype has been reported in GWAS Catalog data as integrated in the GeneCards database (23).

### Construction of the lncRNA/circRNA–miRNA–mRNA regulatory network

**Figure 5:**
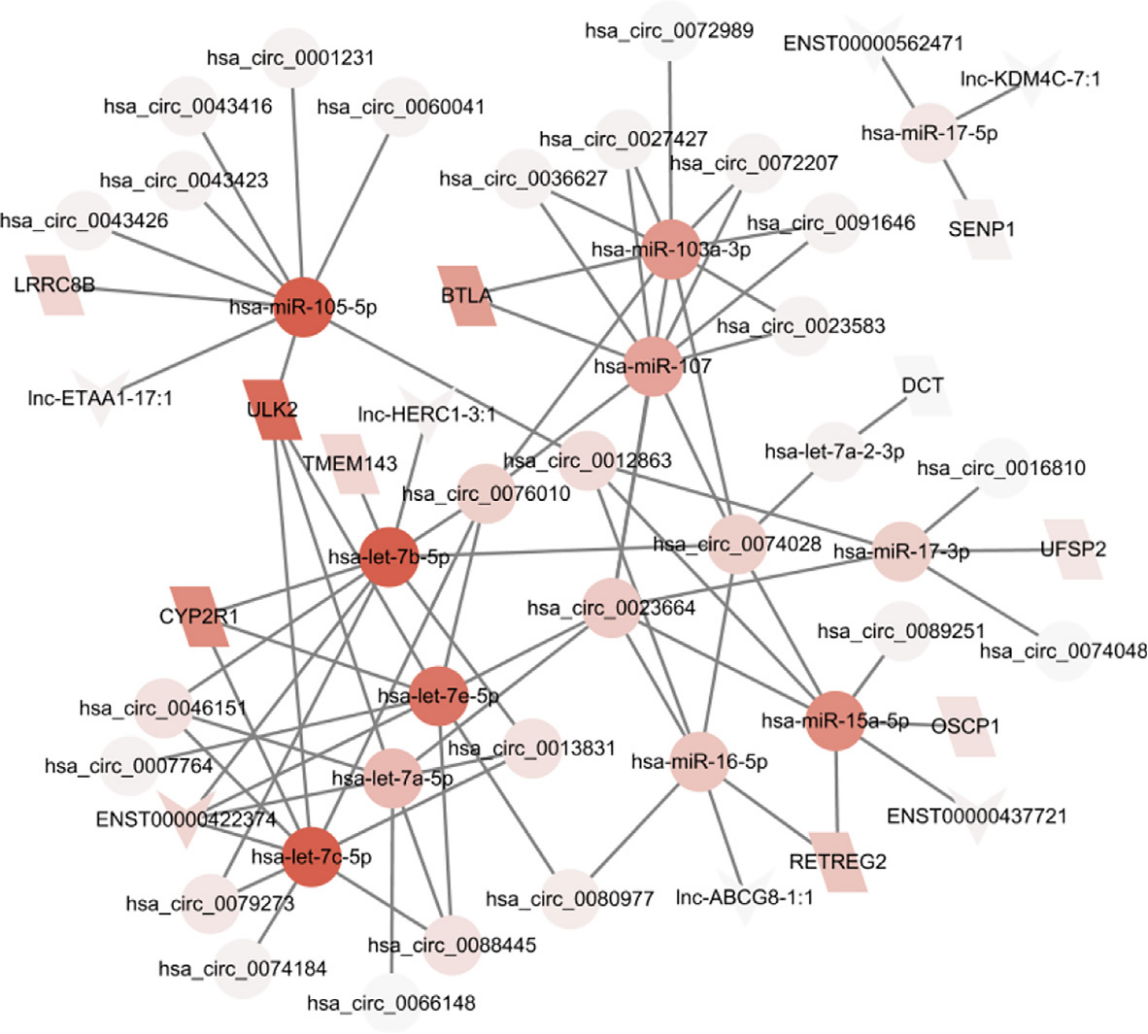
A ceRNA network based on differential genes was constructed which involves 33 lncRNAs/circRNA, 11 mRNAs, and 12 miRNAs.

The ceRNA network can be affected by regulatory molecules. To predict the functions of DELs and DEMs, we constructed a lncRNA/circRNA-miRNA-mRNA ceRNA network with miRNA as the middle regulatory molecule. The network involves 33 lncRNAs/circRNA, 11 mRNAs, and 12 miRNAs (**Figure 5**). Differentially expressed genes can be associated with multiple miRNAs. For example, hsa-miR-105-5p, hsa-miR-7c-5p, and hsa-miR-7b-5p were the top 3 highly connected miRs. ULK2, CYP2R1, and BTLA were the top 3 highly connected mRNAs. hsa_circ_0023664, hsa_circ_0076010, and hsa_circ_0074028 were the top 3 highly connected circRNAs. ENST00000422374 was the highest connected lncRNA. This ceRNA network suggests that lncRNAs and circRNAs can act together as miRNA sponges to influence downstream gene expression and be involved in the development and occurrence of GCs.

### Clinical relevance of ceRNA network hub genes

**Figure 6:**
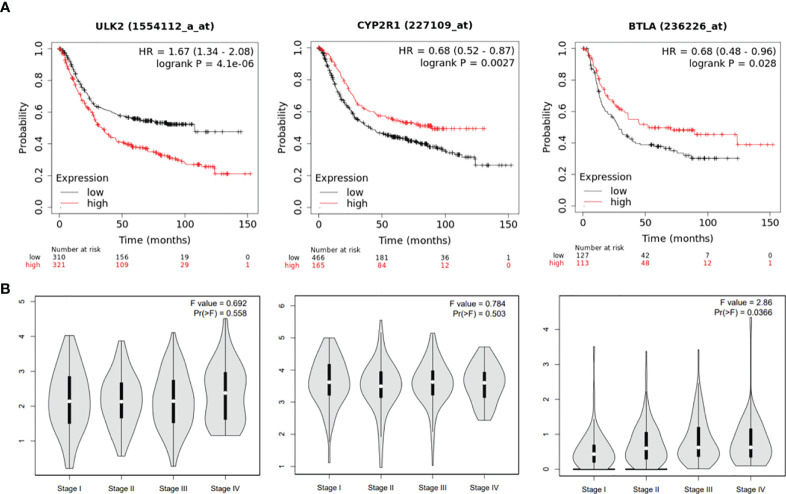
ceRNA network hub genes ULK2, CYP2R1, and BTLA are prognostic in TCGA GC cohort. **(A)** The three genes can separate patients with long and short survival time in GC patients. **(B)** the 3 genes show no significant difference at different GC stages.

**Figure 7:**
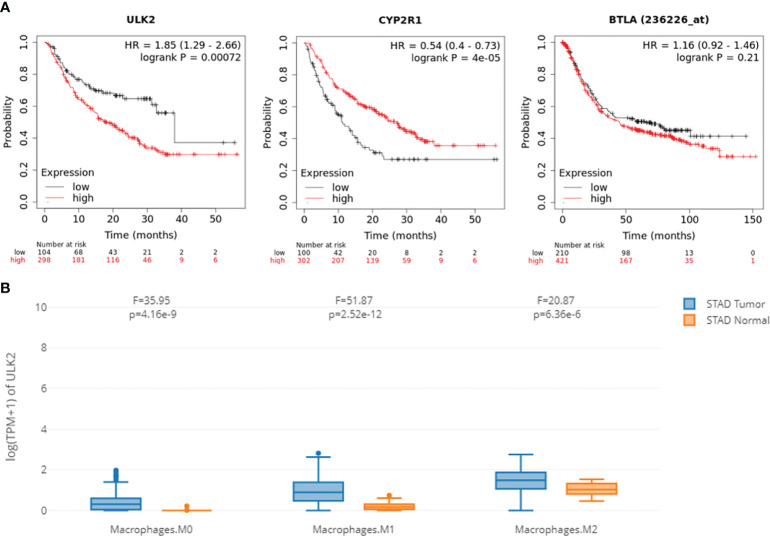
ceRNA network hub genes ULK2, CYP2R1, and BTLA are prognostic in the immunotherapy cohort. **(A)** The three genes can separate patients with long and short survival time from patients with anti-PD-1 treatment. **(B)** Macrophages have significant differential expression when comparing ULK2 between normal and tumor tissues.

To test if the hub genes in the ceRNA network are clinically relevant, we collected GC datasets from public databases and performed survival analysis. Results showed that both ULK2 and CYP2R1 could separate patients with long and short overall survival time, while BTLA only prognosis Lauren diffuse subtype GC (**Figure 6A**). Higher ULK2 indicates shorter survival, while higher CYP2R1 and BTLA indicate longer survival. However, these genes show no significant difference at different GC stages (**Figure 6B**). Paclitaxel treatment could down-regulate ULK2 and up-regulate CYP2R1. As immunotherapy has been applied to many cancer types, we also checked if the hub genes can prognosis patients with anti-PD-1 treatment. Results showed that all three genes can separate patients with long and short survival time (**Figure 7A**), indicating the potential as prognosis biomarkers. The tumor immune microenvironment plays a key role in disease development, therefore, we also analyzed the expression of ULK2 in different tumor immune cell types. Among the 22 CIBERSORT cell types, plasma cell and macrophage had significant differences (P <0.01/22) in abundance when comparing ULK2 between normal and tumor tissues from TCGA dataset. Macrophages had the highest significance (**Figure 7B**), indicating the variation source of ULK2 expression. Furthermore, immune cell-level survival analysis detected M0 and M1 macrophages were negatively correlated with relapse-free survival in GC from TCGA dataset (**Figure S1**).

### Docking analysis of ULK2 and paclitaxel

**Figure 8:**
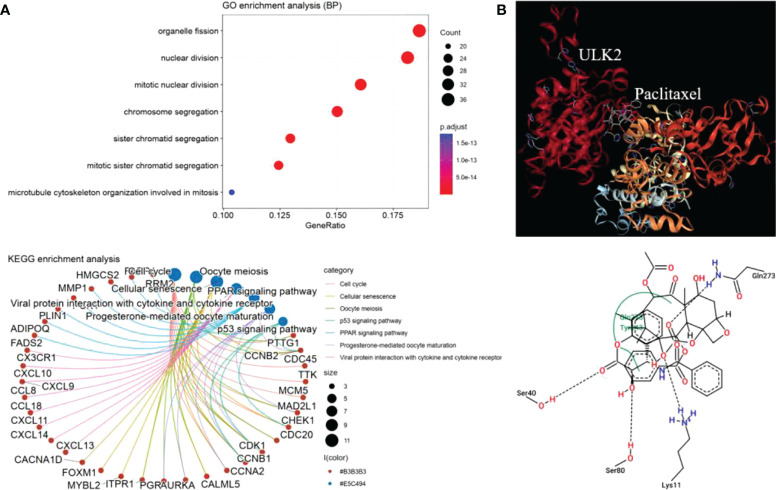
ULK2 functional annotation and docking analysis. **(A)** GO biological process enrichment and KEGG pathway analysis of the top 30 ULK2 similar genes by Pearson correlation analysis. **(B)** Paclitaxel interacts with ULK2 at residues Ser40, Ser80, Lys11, and Gln273.

To understand the mechanisms of ULK2 in paclitaxel treatment, we performed gene correlation analysis and molecule docking. Here, we only chose ULK2 as it had the most significant P value in the previous survival analysis. The top 30 most highly correlated genes with ULK2 were retrieved by Pearson correlation and submitted to clusterProfile for functional enrichment analysis. We found that ULK2 similar genes were significantly involved in mitosis and cell cycle (**Figure 8A**). Thus, paclitaxel may inhibit these pathways through interaction with ULK2. Furthermore, Protein-ligand docking analysis revealed that ULK2 forms a pocket region, 3 hydrogen bonds, and 1 ionic bond for interaction with paclitaxel (**Figure 8B**).

## Discussion

Exosomes are membrane microvesicles derived from endosomes. They carry proteins, lipids, and genetic materials specific to a particular cell. Exosomes may also play an important role in reprogramming recipient cells and in forming disease microenvironments in addition to transferring RNA information (3). Identifying exosomal RNA biomarkers and understanding their functions in exosomes is therefore crucial for understanding diseases. Using ceRNA microarrays, we identified for the first time GC-associated exosomal lncRNAs, circRNAs, and mRNAs response to chemotherapy. As a result of these findings, we have gained new insights into ceRNA networks within exosomes, which can be utilized to identify biomarkers and develop possible therapeutic targets for GC.

Currently, few studies focused on exosomal transcriptome alteration after chemotherapy. In this study, 961 differential RNAs were identified in plasma exosomes in GC patients treated with paclitaxel. Further functional enrichment analysis revealed potential mechanisms of exosomes in paclitaxel treatment. The construction of the ceRNA network suggested the complex regulatory relationships between different RNA molecules. Our study identified hub genes that are involved in disease survival and drug mechanisms. Exosomal mRNA biomarkers in our study, including ULK2, CYP2R1, and BTLA, were also hub mRNAs involved in the responses of GC to paclitaxel. The three genes were all differentially expressed, which were confirmed in TCGA gastric cancer dataset (**Figure S2**).

ULK2 is an upstream autophagy inducer. However, there are contradictory results about the role of ULK2 in cancers. Knockdown of ULK2 expression could significantly induce autophagy, EMT, and migration but suppress proliferation and lung tumor growth in a xenotransplantation model (24). In lung cancer, tumor suppressor miR-137 can reduce the expression of some oncogenic target genes including ULK2 (25). In prostate cancer cells, miR-26b can inhibit autophagy by targeting ULK2, while ULK2 is found not essential for autophagy induction in adult hippocampal neural stem cells following insulin withdrawal (26, 27). In a pan-cancer analysis, different changes in the ULK2 transcript were observed depending on the cancer types (28). ULK2 can be silenced by methylation, which may induce epithelial-mesenchymal transition and transformation to poorly differentiated status in a gastric cancer cell line (29). Using the TCGA dataset, we found SETDB2 might be the upstream regulator of ULK2, which causes dysregulated histone methylation. Our results indicated that ULK2 was down-regulated by paclitaxel, which could be a potential mechanism of action. The contradictory findings identified in studies may be due to its cell-type specific role, diverse roles and loss the context of microenvironment (30, 31). In our findings, ULK2 was not only differentially expressed on cancer cell but also macrophage and plasma cell. What’s more, we had some novel discoveries of the hub mRNAs in exosomes, such as CYP2R1 and BTLA, that haven’t been reported in exosomes but have important functions. CYP2R1 are crucial components of vitamin D-metabolizing enzymes (32). Recent results suggested that high vitamin D concentration was associated with a lower odds ratio of GC in Korean adults (33). In TCGA dataset, CYP2R1 was down-regulated. Our results indicated that CYP2R1 was up-regulated by paclitaxel, and patients with higher CYP2R1 expression had longer survival. Thus, CYP2R1 can be a promising biomarker for prognosis. BTLA is found to be expressed in tumor-infiltrating lymphocytes (TILs) and is often associated with impaired anti-tumor immune response (34). High expression of BTLA was significantly associated with lymph node metastasis and poor prognosis (35). In our results, high BTLA was associated with shorter survival but not significant. Paclitaxel could down-regulate BTLA in exosomes, thus, it is a promising biomarker for prognosis.

In addition, protein-ligand docking was conducted for mechanism research. A complex model with the highest negative minimum ΔG value (-8.24 kcal/mol) among all the studied complexes can be the best possible model. Therefore, the four residues located in the Serine/Threonine kinase catalytic domain of ULK2 may play important role in the chemotherapy response. Further expression correlation analysis confirmed that ULK2 was involved in cell cycle and meiosis, indicating the importance of ULK2 in disease progression as exosomal ULK2 may exert pro-proliferation function when transported to target cells. However, pancreatic cancer derived exosomes cannot induce changes in distant organ metastases in *in vivo* models, but alter early murine gut microbiome (36). The detailed mechanism of ULK2 should be verified experimentally in future.

In the lncRNA/circRNA-miRNA-mRNA network, predicted target miRNAs served as a bridge between lncRNA/circRNA and mRNA. The findings of our study may reveal a new mechanism for EMs. For example, lnc-ETAA1-17:1 and several circRNA could promote the expression of ULK2 by competing with shared target miR-105-5p in exosome, thus may lead to the release of more ULK2 to the receipt cells. It was shown that miR-105-5p overexpression can counter immune escape caused by PD-L1 upregulation in gastric cancer cells (37). Survival analysis also showed that low expression of miR-105-5p was associated with longer OS in TCGA GC patients with a high mutation burden and TCGA immunotherapy cohort (**Figure S3**). Thus, miR-105-5p is a promising biomarker in GC. Further analysis revealed that miR-105-5p could regulate ULK2 by complementary pairings at 3’UTR with an energy -21.9 (**Figure S4**). Besides, genome-wide mutation analysis in the TCGA GC dataset revealed the association between candidate regulatory genes and ULK2 expression (**Figure S5**). The most significant mutation is SETDB2 which is a histone methyltransferase, indicating the possible mechanism of ULK2 in GC. Higher ULK2 expression in GC may be associated with lower histone methylation caused by SETDB2 mutation. Finally, we identified some lncRNA-mRNA pairs most of which are still not reported. Therefore, the study investigates the potential diagnostic and therapeutic benefits of exosomal ceRNA networks by examining the crosstalk between exosome RNAs. The mechanism of the key gene ULK2 was also implicated.

## Data availability statement

The original contributions presented in the study are included in the article/**Supplementary Materials**. Further inquiries can be directed to the corresponding author.

## Ethics statement

The studies involving human participants were reviewed and approved by Ethics Committee of Fujian Provincial Hospital. The patients/participants provided their written informed consent to participate in this study.

## Author contributions

JL and GZ, methodology, investigation, writing – review and editing. DL, methodology, formal analysis, visualization, and writing – original draft. JZ, QC, and LL, visualization, writing – review and editing. ZL, methodology, supervision, and resources. All authors contributed to the article and approved the submitted version.

## Funding

This work was supported in part by the Special Fund for Education and Research of the Provincial Department of Finance, China (grants 00628210101 and 00628210103).

## Conflict of interest

The authors declare that the research was conducted in the absence of any commercial or financial relationships that could be construed as a potential conflict of interest.

## Publisher’s note

All claims expressed in this article are solely those of the authors and do not necessarily represent those of their affiliated organizations, or those of the publisher, the editors and the reviewers. Any product that may be evaluated in this article, or claim that may be made by its manufacturer, is not guaranteed or endorsed by the publisher.

## Supplementary material

Supplementary Figure 1 M0 and M1 macrophages were negatively correlated with relapse-free survival in GC

Supplementary Figure 2 ceRNA network hub genes ULK2, CYP2R1, and BTLA were differentially expressed in TCGA GC dataset

Supplementary Figure 3 Low expression of miR-105-5p was associated with longer OS in TCGA GC patients with a high mutation burden and TCGA immunotherapy cohort

Supplementary Figure 4 miR-105-5p could regulate ULK2 by complementary pairings at 3’UTR with an energy -21.9

Supplementary Figure 5 Genome-wide mutation analysis in the TCGA GC dataset revealed the association between five candidate regulatory genes and ULK2 expression

## References

[1] Hartgrink HH Jansen EP van Grieken NC van de Velde CJ . Gastric cancer. Lancet (2009) 374:477–90. doi: 10.1016/S0140-6736(09)60617-6 PMC4613761 19625077

[2] Smyth EC Nilsson M Grabsch HI van Grieken NC Lordick F . Gastric cancer. Lancet (2020) 396:635–48. doi: 10.1016/S0140-6736(20)31288-5 32861308

[3] Dai J Su Y Zhong S Cong L Liu B Yang J . Exosomes: key players in cancer and potential therapeutic strategy. Signal Transduct Target Ther (2020) 5:145. doi: 10.1038/s41392-020-00261-0 32759948 PMC7406508

[4] Statello L Guo CJ Chen LL Huarte M . Gene regulation by long non-coding RNAs and its biological functions. Nat Rev Mol Cell Biol (2021) 22:96–118. doi: 10.1038/s41580-020-00315-9 33353982 PMC7754182

[5] Verduci L Tarcitano E Strano S Yarden Y Blandino G . CircRNAs: role in human diseases and potential use as biomarkers. Cell Death Dis (2021) 12:468. doi: 10.1038/s41419-021-03743-3 33976116 PMC8113373

[6] He L Hannon GJ . MicroRNAs: small RNAs with a big role in gene regulation. Nat Rev Genet (2004) 5:522–31. doi: 10.1038/nrg1379 15211354

[7] Xu Q Jia X Wu Q Shi L Ma Z Ba N . Esomeprazole affects the proliferation, metastasis, apoptosis and chemosensitivity of gastric cancer cells by regulating lncRNA/circRNA-miRNA-mRNA ceRNA networks. Oncol Lett (2020) 20:329. doi: 10.3892/ol.2020.12193 33101498 PMC7577076

[8] Yang H Yang S Shen H Wu S Ruan J Lyu G . Construction of the amniotic fluid-derived exosomal ceRNA network associated with ventricular septal defect. Genomics (2021) 113:4293–302. doi: 10.1016/j.ygeno.2021.11.003 34758360

[9] Weaver BA . How taxol/paclitaxel kills cancer cells. Mol Biol Cell (2014) 25:2677–81. doi: 10.1091/mbc.e14-04-0916 PMC4161504 25213191

[10] Sakamoto J Matsui T Kodera Y . Paclitaxel chemotherapy for the treatment of gastric cancer. Gastric Cancer (2009) 12:69–78. doi: 10.1007/s10120-009-0505-z 19562460

[11] Zhang L Xu X Yang R Chen J Wang S Yang J . Paclitaxel attenuates renal interstitial fibroblast activation and interstitial fibrosis by inhibiting STAT3 signaling. Drug Des Devel Ther (2015) 9:2139–48. doi: 10.2147/DDDT.S81390 PMC4404961 25931810

[12] Wu T Hu E Xu S Chen M Guo P Dai Z . clusterProfiler 4.0: A universal enrichment tool for interpreting omics data. Innovation (Camb) (2021) 2:100141. doi: 10.1016/j.xinn.2021.100141 34557778 PMC8454663

[13] Betel D Wilson M Gabow A Marks DS Sander C . The microRNA.org resource: targets and expression. Nucleic Acids Res (2008) 36:D149–153. doi: 10.1093/nar/gkm995 PMC2238905 18158296

[14] Chen Y Wang X . miRDB: An online database for prediction of functional microRNA targets. Nucleic Acids Res (2020) 48:D127–31. doi: 10.1093/nar/gkz757 PMC6943051 31504780

[15] Shannon P Markiel A Ozier O Baliga NS Wang JT Ramage D . Cytoscape: A software environment for integrated models of biomolecular interaction networks. Genome Res (2003) 13:2498–504. doi: 10.1101/gr.1239303 PMC403769 14597658

[16] Team RC . R: A language and environment for statistical computing. (Vienna, Austria: CRAN) (2016).

[17] Clough E Barrett T . The gene expression omnibus database. Methods Mol Biol (2016) 1418:93–110. doi: 10.1007/978-1-4939-3578-9_5 27008011 PMC4944384

[18] Liu J Lichtenberg T Hoadley KA Poisson LM Lazar AJ Cherniack AD . An integrated TCGA pan-cancer clinical data resource to drive high-quality survival outcome analytics. Cell (2018) 173:400–416 e411. doi: 10.1016/j.cell.2018.02.052 29625055 PMC6066282

[19] Burley SK Bhikadiya C Bi C Bittrich S Chen L Crichlow GV . RCSB protein data bank: powerful new tools for exploring 3D structures of biological macromolecules for basic and applied research and education in fundamental biology, biomedicine, biotechnology, bioengineering and energy sciences. Nucleic Acids Res (2021) 49:D437–51. doi: 10.1093/nar/gkaa1038 PMC7779003 33211854

[20] Grosdidier A Zoete V Michielin O . SwissDock, a protein-small molecule docking web service based on EADock DSS. Nucleic Acids Res (2011) 39:W270–277. doi: 10.1093/nar/gkr366 PMC3125772 21624888

[21] Schoning-Stierand K Diedrich K Fahrrolfes R Flachsenberg F Meyder A Nittinger E . ProteinsPlus: interactive analysis of protein-ligand binding interfaces. Nucleic Acids Res (2020) 48:W48–53. doi: 10.1093/nar/gkaa235 PMC7319454 32297936

[22] Stierand K Maass PC Rarey M . Molecular complexes at a glance: automated generation of two-dimensional complex diagrams. Bioinformatics (2006) 22:1710–6. doi: 10.1093/bioinformatics/btl150 16632493

[23] Safran M Dalah I Alexander J Rosen N Iny Stein T Shmoish M . GeneCards version 3: the human gene integrator. Database (Oxford) (2010), 2010: baq020 doi: 10.1093/database/baq020 20689021 PMC2938269

[24] Kim YH Baek SH Kim EK Ha JM Jin SY Lee HS . Uncoordinated 51-like kinase 2 signaling pathway regulates epithelial-mesenchymal transition in A549 lung cancer cells. FEBS Lett (2016) 590:1365–74. doi: 10.1002/1873-3468.12172 27062295

[25] Zhang B Ma Z Li X Zhang C Shao Y Liu Z . Tanshinones suppress non-small cell lung cancer through up-regulating miR-137. Acta Biochim Biophys Sin (Shanghai) (2016) 48:768–70. doi: 10.1093/abbs/gmw053 27297636

[26] John Clotaire DZ Zhang B Wei N Gao R Zhao F Wang Y . MiR-26b inhibits autophagy by targeting ULK2 in prostate cancer cells. Biochem Biophys Res Commun (2016) 472:194–200. doi: 10.1016/j.bbrc.2016.02.093 26920049

[27] Ryu HY Kim LE Jeong H Yeo BK Lee JW Nam H . GSK3B induces autophagy by phosphorylating ULK1. Exp Mol Med (2021) 53:369–83. doi: 10.1038/s12276-021-00570-6 PMC8080724 33654220

[28] Kumar M Papaleo E . A pan-cancer assessment of alterations of the kinase domain of ULK1, an upstream regulator of autophagy. Sci Rep (2020) 10:14874. doi: 10.1038/s41598-020-71527-4 32913252 PMC7483646

[29] Motoo I Nanjo S Ando T Yamashita S Ushijima T Yasuda I . Methylation silencing of ULK2 *via* epithelial-mesenchymal transition causes transformation to poorly differentiated gastric cancers. Gastric Cancer (2022) 25:325–35. doi: 10.1007/s10120-021-01250-0 34554345

[30] Demeter A Romero-Mulero MC Csabai L Olbei M Sudhakar P Haerty W . ULK1 and ULK2 are less redundant than previously thought: computational analysis uncovers distinct regulation and functions of these autophagy induction proteins. Sci Rep (2020) 10:10940. doi: 10.1038/s41598-020-67780-2 32616830 PMC7331686

[31] McAlpine F Williamson LE Tooze SA Chan EY . Regulation of nutrient-sensitive autophagy by uncoordinated 51-like kinases 1 and 2. Autophagy (2013) 9:361–73. doi: 10.4161/auto.23066 PMC3590256 23291478

[32] Wen J Li J Liang X Wang A . Association of polymorphisms in vitamin d-metabolizing enzymes DHCR7 and CYP2R1 with cancer susceptibility: A systematic review and meta-analysis. Dis Markers (2021) 2021:6615001. doi: 10.1155/2021/6615001 34093899 PMC8164542

[33] Kwak JH Paik JK . Vitamin d status and gastric cancer: A cross-sectional study in koreans. Nutrients (2020) 12:2004. doi: 10.3390/nu12072004 PMC7400919 32640566

[34] Ning Z Liu K Xiong H . Roles of BTLA in immunity and immune disorders. Front Immunol (2021) 12:654960. doi: 10.3389/fimmu.2021.654960 33859648 PMC8043046

[35] Lan X Li S Gao H Nanding A Quan L Yang C . Increased BTLA and HVEM in gastric cancer are associated with progression and poor prognosis. Onco Targets Ther (2017) 10:919–26. doi: 10.2147/OTT.S128825 PMC5317317 28243127

[36] Charles Jacob HK Signorelli R Charles Richard JL Kashuv T Lavania S Middleton A . Identification of novel early pancreatic cancer biomarkers KIF5B and SFRP2 from "first contact" interactions in the tumor microenvironment. J Exp Clin Cancer Res (2022) 41:258. doi: 10.1186/s13046-022-02425-y 36002889 PMC9400270

[37] Miliotis C Slack FJ . miR-105-5p regulates PD-L1 expression and tumor immunogenicity in gastric cancer. Cancer Lett (2021) 518:115–26. doi: 10.1016/j.canlet.2021.05.037 PMC8355212 34098061

