# Supplementary material for: Analysis of exosomal competing endogenous RNA network response to paclitaxel treatment reveals key genes in advanced gastric cancer
Source: Front Oncol. 2022 Oct 25;12:1027748. doi: 10.3389/fonc.2022.1027748 (PMC9640578; doi:10.3389/fonc.2022.1027748)
Supplement: Supplementary Figure 1 — M0 and M1 macrophages were negatively correlated with relapse-free survival in GC [file DataSheet_1.docx]

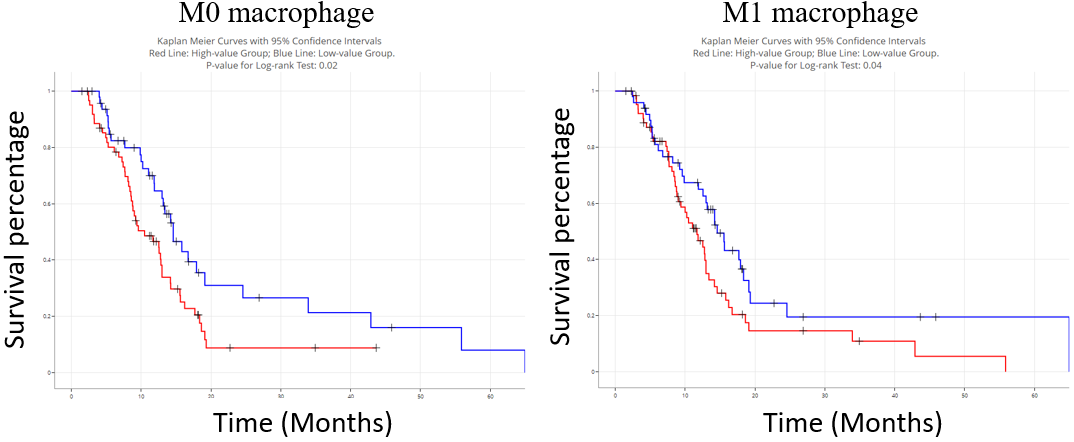


**SUPPLEMENTARY FIGURE 1** M0 and M1 macrophages were negatively correlated with relapse-free survival in GC


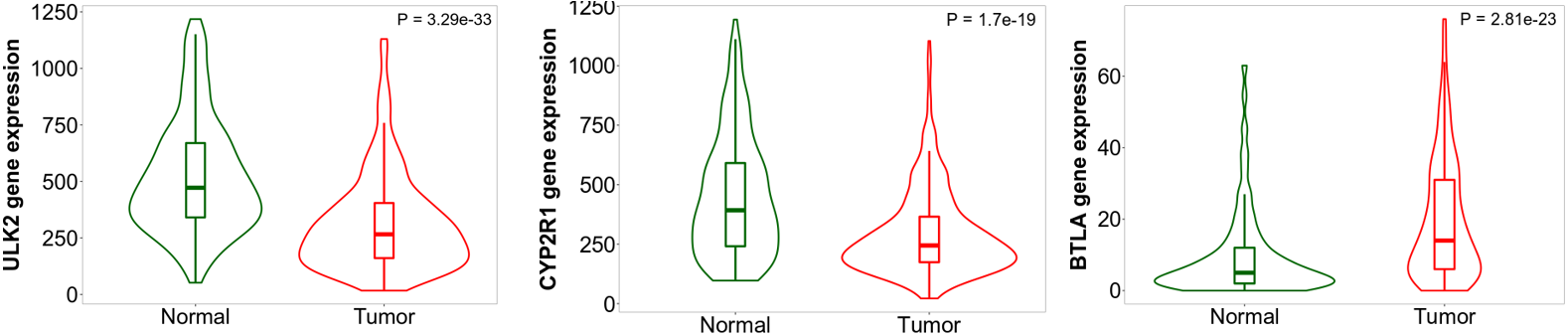


**SUPPLEMENTARY FIGURE 2** ceRNA network hub genes ULK2, CYP2R1, and BTLA were differentially expressed in TCGA GC dataset


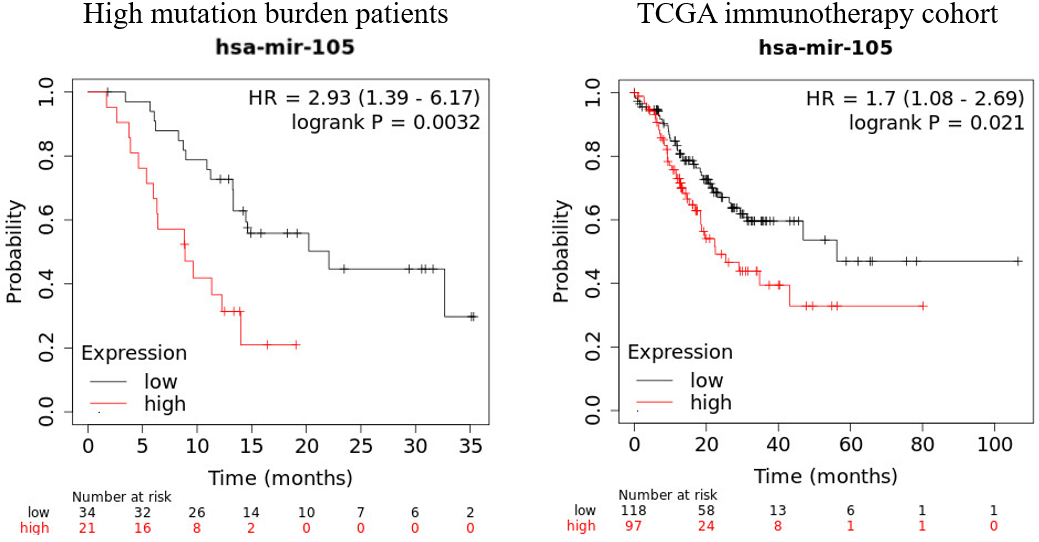


**SUPPLEMENTARY FIGURE 3** Low expression of miR-105-5p was associated with longer OS in TCGA GC patients with a high mutation burden and TCGA immunotherapy cohort


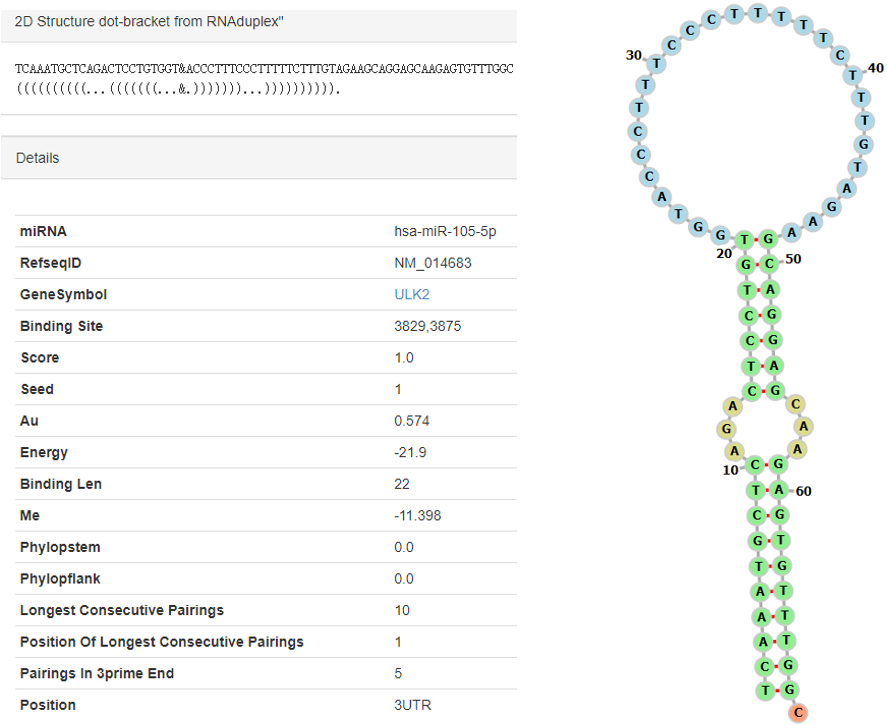


**SUPPLEMENTARY FIGURE 4** miR-105-5p could regulate ULK2 by complementary pairings at 3’UTR with an energy -21.9


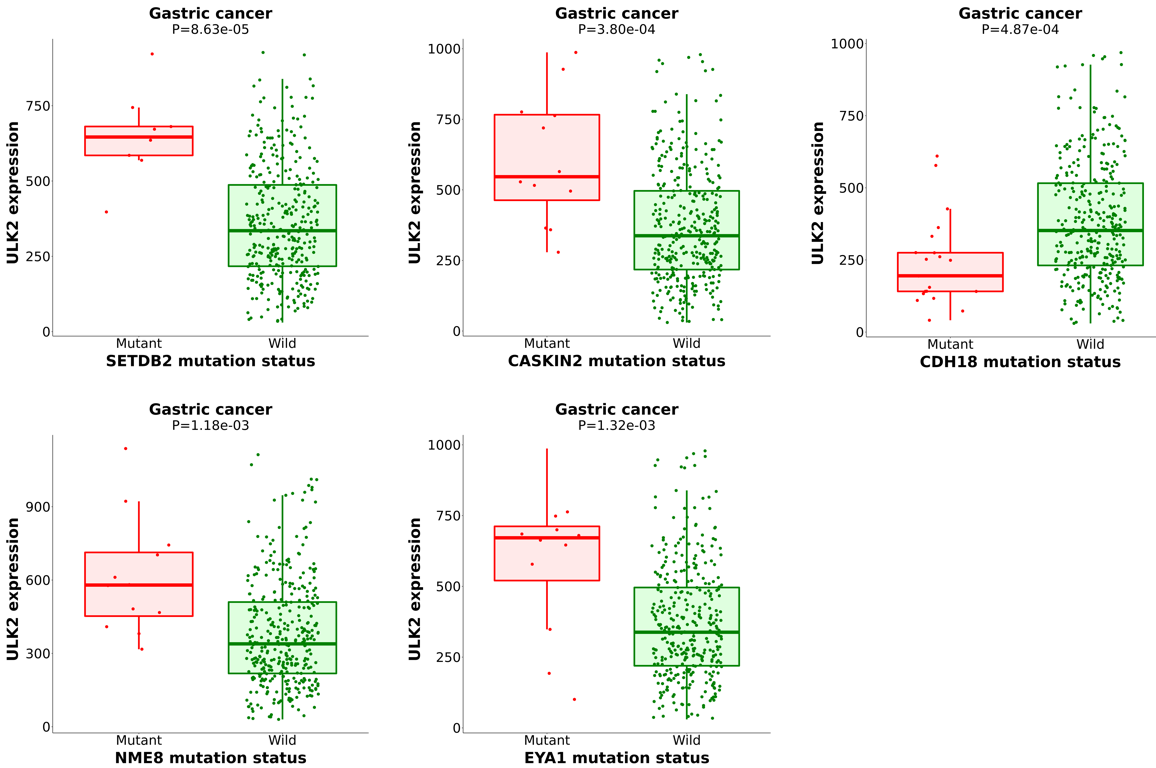


**SUPPLEMENTARY FIGURE 5** Gnome-wide mutation analysis in the TCGA GC dataset revealed the association between five candidate regulatory genes and ULK2 expression
